# Long-term Chikungunya sequelae and quality of life 2.5 years post-acute disease in a prospective cohort in Curaçao

**DOI:** 10.1371/journal.pntd.0010142

**Published:** 2022-03-01

**Authors:** Churnalisa Doran, Jelte Elsinga, Ante Fokkema, Kevin Berenschot, Izzy Gerstenbluth, Ashley Duits, Norediz Lourents, Yaskara Halabi, Johannes Burgerhof, Ajay Bailey, Adriana Tami

**Affiliations:** 1 University of Groningen, University Medical Center Groningen, Department of Medical Microbiology and Infection Prevention, Groningen, The Netherlands; 2 Curaçao Biomedical and Health Research Institute, Willemstad, Curaçao; 3 Department of Infectious Diseases, Public Health Service Amsterdam, Amsterdam, The Netherlands; 4 University of Groningen, Groningen, The Netherlands; 5 Department of Epidemiology and Research, Medical and Public Health Service Curaçao, Willemstad, Curaçao; 6 Red Cross Blood Bank Foundation, Willemstad, Curaçao; 7 University of Groningen, University Medical Center Groningen, Department of Epidemiology, Groningen, The Netherlands; 8 Department of Human Geography and Spatial Planning, University of Utrecht, Utrecht, The Netherlands; Universidade do Estado do Para: Universidade do Estado do Para, BRAZIL

## Abstract

**Background:**

Little is known about the persistence and impact of non-rheumatic symptoms after acute chikungunya disease. We have studied the clinical presentation and long-term impact of rheumatic and non-rheumatic symptoms on health related quality of life (QoL) 2.5 years after disease onset. Additionally, the validity of the Curaçao Long-Term Chikungunya Sequelae (CLTCS) score in classifying disease severity over time was evaluated.

**Methodology/Principal findings:**

This prospective cohort study followed 248 chikungunya patients. Symptoms and SF-36 QoL were evaluated during baseline and follow-up at 2.5 years using questionnaires. Chikungunya disease status was classified using the CLTCS-score. At 2.5 years after disease onset patients were classified as being recovered (43%), mildly (35%) or highly (22%) affected. In comparison to mildly affected, highly affected patients reported the highest prevalence of ongoing rheumatic and non-rheumatic/psychological symptoms, with increased prevalence of arthralgia in the lower extremities (p = .01) and fatigue (p = .049) over time, and higher pain intensity (p < .001). Compared to mildly affected, being highly affected was associated with weakness in the lower extremities (OR: 1.90; CI: 1.29–2.80, p = .001) and worsened physical and mental QoL impairment.

**Conclusions:**

Patients are both physically and psychologically affected by rheumatic and non-rheumatic symptoms of long-term chikungunya disease.

The CLTCS-score is an easy to use instrument for classifying long-term chikungunya disease severity and impact and can facilitate health care providers in identifying highly affected patients who are prone to develop severe QoL impairment. Highly affected patients are recommended to be treated in a multidisciplinary setting to improve physical and psychological functioning, and QoL.

## Author summary

Chikungunya disease manifestation is characterized by a sudden onset of non-rheumatic flu-like symptoms and debilitating rheumatic symptoms in the acute phase. Little is known about the persistence of non-rheumatic symptoms after acute disease. In this prospective cohort study we investigated the clinical manifestations and persistence of baseline rheumatic and non-rheumatic/psychological symptoms and their impact on health related quality of life (QoL) 2.5 years after disease onset. Moreover, we investigated the performance of the classification instrument the Curaçao Long-Term Chikungunya Sequelae (CLTCS) score in classifying chikungunya disease severity over time. We found that 57% of the patients were still affected 2.5 years after disease onset. An increase of mainly recurrent rheumatic and non-rheumatic/psychological symptoms including fatigue, insomnia, sombreness, and loss of vitality were reported, with a significantly higher symptom recurrence and pain intensity reported by highly affected patients. In addition, QoL assessment indicates that the disease burden impaired the physiological well-being of these patients. We also showed that the CLTCS-score can be used to easily identify highly affected patients. This study demonstrates that disease severity increases non-rheumatic symptoms and subsequent physiological impairments and suggests a multidisciplinary treatment approach to treat the psychological effects of long-term chikungunya disease.

## Introduction

Chikungunya disease is caused by the arthritogenic chikungunya virus (CHIKV). The virus is an arthropod-borne pathogen, transmitted by an infected day-biting female *Aedes* mosquito [1]. Chikungunya disease starts with acute disease (7–10 days), characterized by an abrupt onset of non-rheumatic and rheumatic symptoms, including high fever, headache, nausea, fatigue, maculopapular skin rash, intense myalgia, and severe debilitating polyarthralgia [2, 3].

Some patients with acute disease recover fully, but the severe and debilitating polyarthralgia may persist and progress to sub-acute (<3 months), or chronic disease (>3 months—years), characterized by either recurrent or constant symptoms [4–7], affecting everyday activities and causing impaired life satisfaction and health related quality of life (QoL) [8–13].

Besides the debilitating polyarthralgia, little is known about the persistence and impact of non-rheumatic symptoms during post-acute disease. Nevertheless, research regarding this topic remains scarce, given the fact that chikungunya was previously often viewed as a benign and self-limiting disease [14], with the majority of symptoms resolving in 7–10 days in many individuals with infrequent severe long-term manifestation [15]. To better understand the clinical manifestation of long-term chikungunya disease and its effects on the lives of patients, periodic assessment of disease progression and impact on QoL is necessary. The present study addresses these knowledge gaps by describing the impact of rheumatic symptoms and a wide range of non-rheumatic symptoms on QoL following chikungunya disease onset.

In December 2013, the introduction of CHIKV became evident in the Americas [16]. By June-July 2014 it reached Curaçao, infecting approximately 30–50% of the 150.000 inhabitants, causing a major long-term impact on QoL [8]. In 2015, we established a longitudinal cohort study of chikungunya patients in Curaçao.

In the baseline study, the Curaçao Long-Term Chikungunya Sequelae (CLTCS) score was developed to classify the impact of chikungunya on individuals [8]. The present study evaluates disease progression and impact of long-term sequelae on QoL, in patients with different levels of chikungunya disease status based on the CLTCS score, in comparison to our baseline study.

The study had two aims: 1) to investigate the prevalence, nature and severity of symptoms, QoL, and explanatory variables of chronic chikungunya disease status >2.5 years after disease onset; 2) to compare the disease evolution from baseline to >2.5 years after disease onset, using the CLTCS-score to characterize chronic chikungunya disease.

## Methods

### Ethics statement

The study was approved by the Medical Ethical Board of the Saint Elisabeth Hospital in Curaçao (Reference number: 2015–002). Written informed consent was obtained from each patient after explaining all the study objectives. The study includes the STROBE statement (S1 Strobe cheklist).

### Setting

Curaçao is a 444 square kilometres autonomous island state within the Kingdom of the Netherlands, located in the southern Caribbean Sea, 65 km north of Venezuela [17].

### Study design and population

This follow-up study is part of a longitudinal prospective cohort of laboratory confirmed adult chikungunya patients (age ≥ 18 years), selected in 2015 through 14 health centres representative of the population of Curaçao. In the present study, a follow-up survey was performed in 2017 on the same patients previously recruited during the 2015 baseline study. Here, we compare the findings in 2017 versus those in 2015 in order to understand the long-term impact of chikungunya in affected individuals.

### Baseline assessment

During baseline 334 laboratory confirmed chikungunya patients were enrolled into a cohort study. Between June and July 2015, the baseline study assessed patients 3–16 months after chikungunya disease onset (henceforth referred to as 2015). A questionnaire administered by trained local interviewers contained pre-coded and open questions on socio-demographic characteristics, comorbidities, date of disease onset, duration of symptoms, presence of (rheumatic) symptoms and impact on QoL (SF-36). The patients of the cohort study were classified into the long-term chikungunya disease severity categories recovered, mildly affected, or highly affected, based on self-declared conditions using the CLTCS-score. Data collection and analyses in the baseline study were described in detail elsewhere [8].

### Follow-up and clinical presentation

Between February-April 2017 (henceforth referred to as 2017), all patients included in the baseline study were contacted by telephone for a follow-up assessment. Patients who did not wish to participate or could not be reached after three failed telephone contact attempts were excluded from the study. After consent, interviews took place at home or work to ensure maximum accessibility to the study and to minimize attrition bias.

The questionnaire in the follow-up study of 2017 contained the same questions as those used in the baseline study (in 2015), and additional questions. The questionnaire was administered in the native language Papiamento, Spanish, Dutch, or English by trained interviewers. We assessed the duration of symptoms and pre-coded questions on the presence of rheumatic and non-rheumatic symptoms at the time of the follow-up interview. The non-rheumatic symptoms headache, loss of appetite, sore throat, chills, conjunctivitis, and sensitivity to light were added to the 2017 questionnaire. Patients answering ‘somewhat’ or ‘yes’ to symptoms during follow-up were considered symptomatic. Patients could indicate whether the persistent symptoms were ‘recurrent’, or ‘constant’ in nature. Recurrent was defined as relapsing symptoms, with periods of relief and subsequent reoccurrence, but present at the time of interview. Constant was defined as unremitting symptoms, without periods of relief, since baseline. Patients experiencing recurrent symptoms were asked to indicate the relapse frequency of symptoms recurrence. To evaluate progression of pain, the Chikungunya Disease Intensity (CDI) was assessed by using a Visual Analogue Scale (VAS) score (0 = no complaints at all; 10 = worst possible complaints I can imagine) during 5 periods in time: 1) beginning of disease onset until 14th day; 2) 14th day until 6 months after disease onset; 3) 6 months until 1 year; 4) 1 year until 2 years; 5) 2 years until 3 years after disease onset. In 2017, possible newly developed comorbidities were not measured.

### Long-term chikungunya disease status

The patients’ long-term chikungunya disease status was re-assessed at the time of the 2017 interview and reclassified into the chikungunya disease severity categories recovered, mildly affected, or highly affected based on their self-declaration, by using the CLTCS-score [8]. The CLTCS-score assesses the perceived chikungunya disease status, taking the broad clinical presentation and the recurrent and/or constant nature of symptoms into account.

The CLTCS-score comprises of 4 items to assess the long-term chikungunya disease status. In each of its 4 items, the chikungunya disease severity is rated on a five-level scale (Table 1). The scores of items 1 and 2 are recoded and after recoding the scores of all 4 items are summed up to compute a chikungunya disease severity score and categorized into recovered (score = 4), mildly affected (score = 5–12), and highly affected (score = 13–20). Cronbach’s Alpha Test was used to assess the internal consistency, or reliability of the CLTCS-score items and yielded a high score of 0.891.

**Table 1:** CLTCS-score assessment instrument.

To what extent do you agree with the following statements:	Totally disagree	Disagree	Neutral	Agree	Totally agree
1. I am fully functional again after chikungunya	1	2	3	4	5
2. I don’t have any more complaints of chikungunya	1	2	3	4	5
3. I still feel the effects of chikungunya every day	1	2	3	4	5
4. The chikungunya effects seems to return again and again in my case	1	2	3	4	5

Reproduced and modified from Long-term Chikungunya Sequelae in Curaçao: Burden, Determinants, and a Novel Classification Tool, J. Elsinga et al., *J Infect Dis*. *2017 Sep 1;216(5)*:*573–581* [8]. Copyright (2017) by Oxford University Press on behalf of the Infectious Diseases Society of America (IDSA). With permission of Oxford University Press. Recode the scores of statements 1 and 2 into 5 = 1; 4 = 2; 3 = 3; 2 = 4; 1 = 5. After recoding, the scores of all 4 items are summed up. The total obtained score is categorized into the chikungunya disease status recovered (score = 4), mildly affected (score = 5–12), and highly affected (score = 13–20).

### Health-related QoL

The QoL was measured using the 36-item short-form health survey (SF-36) assessment. The SF-36 assesses the full range of health status and well-being, covering eight health and two summary domains, namely, physical (PCS) and mental (MCS) health components [18, 19]. The PCS and MCS minimum clinically important difference (MCID) was defined as 2.5–5 point change criterion for improvement [20].

### Data analysis

SPSS Data Entry Station (SPSS Inc., version 23.0, Chicago, Illinois) was used for data entry and analyses. To follow disease progression, clinical presentation, and impact on QoL, the 2015 and 2017 time points data were analysed to look at individual changes, and compared between the recovered, mildly affected, and highly affected patients after disease status stratification. To evaluate the fluctuation of recurrent symptoms and subsequently possible change of disease status classification over time, the disease status classification (recovered, mildly, or highly affected) and the prevalence of clinical symptoms reported in 2015 and 2017 were compared, with 2015 as baseline. In addition, to evaluate disease evolution and association between the prevalence, nature, and relapse frequency of symptoms, and QoL with disease status 2.5 years after disease onset, the reassessed disease status classification in 2017 was further used in the analysis. The comorbidities that were reported in 2015 were used in the analyses. The primary outcomes for the analyses were self-reported clinical presentation, disease severity according to the CLTCS-score, and QoL at follow-up.

Descriptive analysis was performed using means ± standard deviations (SD) or medians with interquartile ranges (IQR) for continuous variables, and frequencies and percentages for categorical variables. The valid percentages instead of percentages were used, if data was missing. The 2015 and re-assessed 2017 disease status were compared using the McNemar-Bowker test.

The association between categorical variables and disease status were assessed by the chi-square or Fisher’s exact test, matched binary outcomes were compared by McNemar test. The means of normally distributed continuous variables were compared using the Student’s t-test or analysis of variance, and for skewed distributions, the Mann-Whitney or Kruskal-Wallis tests.

Univariate analyses were performed to explore the associations between long-term chikungunya disease status in 2017 and the explanatory variables such as socio-demographic characteristics and symptoms at follow-up, and included in a multivariate binary logistic regression model if their two-sided P-value was ≤ .1 using backward stepwise selection, to describe the characteristics and symptoms independently associated with chikungunya disease status classification (recovered vs. affected and mildly affected vs. highly affected) in 2017 as outcome. In the binary logistic regression analysis gender and age were fitted irrespective of the P-value. Statistically significant (p ≤ .05) characteristics were kept in the final model, if no collinearity was observed. The magnitude of association was measured using the odds ratio (OR) and the 95% confidence interval (CI).

## Results

A total of 248 patients identified during baseline in 2015 consented to participate in the follow-up study (response rate 82%), the other 56 patients were lost to follow-up (S1 Table). The time between the follow-up interview and disease onset was approximately 29 months (2.5 years).

### Long-term disease status and characteristics study population

In 2015, the percentage of patients that were classified by the CLTCS-score as recovered, mildly affected, or highly affected based on self-declared conditions were 93 (37%), 84 (34%), and 71 (29%), respectively. In 2017, more patients were classified as being recovered or mildly affected and less patients as highly affected compared to 2015.

**Fig 1:**
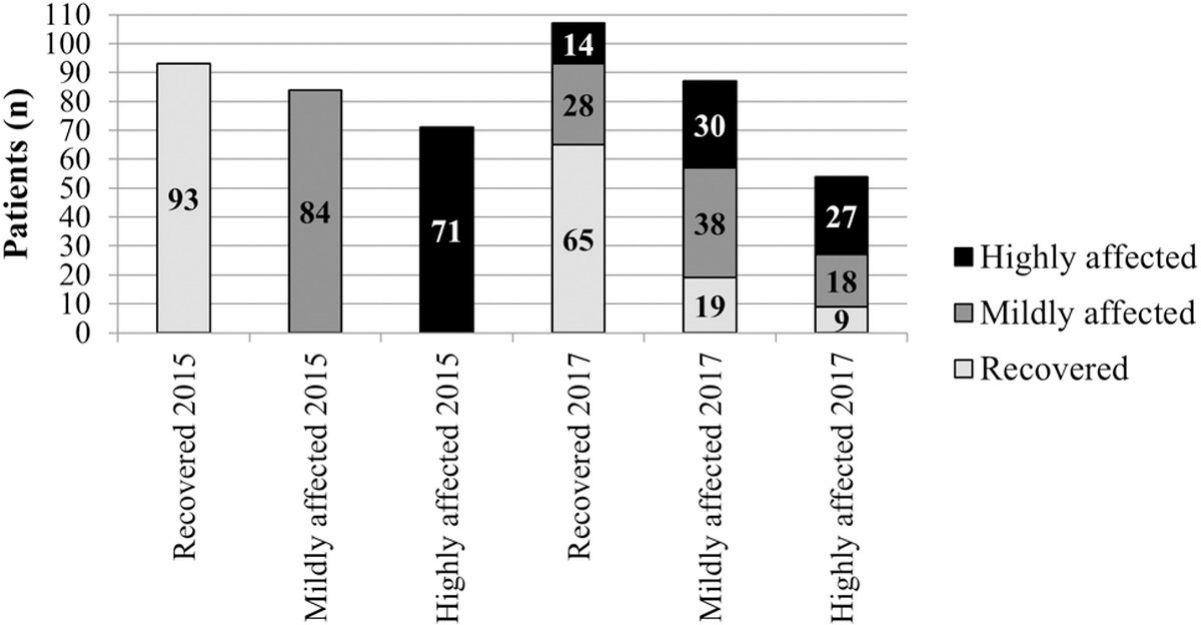
The Curaçao long-term Chikungunya sequelae classification score of the cohort (n = 248). The chikungunya disease status of the cohort during baseline in 2015 and follow-up in 2017 using the Curaçao long-term chikungunya sequelae score. The colors refer to the chikungunya disease status in 2015. Groups were compared using McNemar-Bowker test, two-sided P-value = .12.

Although the change of disease status was not statistically significant, from the 93 patients that were classified as being recovered in 2015, 30% were classified as either mildly (n = 19) or highly (n = 9) affected in 2017. In addition, 38 (45%) of the mildly affected patients remained mildly affected, while 27 (38%) of the highly affected remained highly affected. In total, 107 (43%), 87 (35%), and 54 (22%) of the patients were classified as recovered, mildly affected, or highly affected, respectively, in 2017 (Fig 1).

The socio-demographic characteristics 2.5 years after disease onset and pre-existing comorbidities of the patients based on the 2017 disease status classification are summarized in Table 2.

**Table 2:** Socio-demographic characteristics of the cohort (n = 248), stratified by chronic chikungunya disease status in 2017.

	Total		Recovered		Mildly affected		Highly affected		
	(n = 248)		(n = 107)		(n = 87)		(n = 54)		
	n	(%)	n	(%)	n	(%)	N	(%)	P-value^a^
**Gender**									.11
Female	181	(73.0)	71	(66.4)	69	(79.3)	41	(75.9)	
Male	67	(27.0)	36	(33.6)	18	(20.7)	13	(24.1)	
**Age (years)**									.23
20–40	44	(17.7)	25	(23.4)	14	(16.1)	5	(9.3)	
41–60	127	(51.2)	49	(45.8)	46	(52.9)	32	(59.3)	
≥61	77	(31.0)	33	(30.8)	27	(31.0)	17	(31.5)	
**Education** ^b^									.31
Illiterate/primary school	58	(23.4)	26	(24.3)	16	(18.4)	16	(29.6)	
Secondary school	87	(35.1)	36	(33.6)	33	(37.9)	18	(33.3)	
Intermediate vocational education	65	(26.2)	25	(23.4)	29	(33.3)	11	(20.4)	
University (of applied sciences)	38	(15.3)	20	(18.7)	9	(10.3)	9	(16.7)	
**Occupation** ^b^ ^c^									.36
Unemployed/student/housewife/voluntary	47	(19.0)	16	(15.1)	21	(24.1)	10	(18.5)	
Paid job (domestic or manual)	106	(42.9)	51	(48.1)	32	(36.8)	23	(42.6)	
Paid job (not domestic nor manual)	50	(20.2)	21	(19.8)	21	(24.1)	8	(14.8)	
Retired	44	(17.8)	18	(17.0)	13	(14.9)	13	(24.1)	
**Monthly income** ^b^ ^d^ ^e^									.30
0–999 ANG	23	(9.5)	10	(9.6)	7	(8.2)	6	(11.1)	
1000–2499 ANG	97	(39.9)	42	(40.4)	34	(40.0)	21	(38.9)	
2500–4999 ANG	92	(37.9)	33	(31.7)	35	(41.2)	24	(44.4)	
≥5000 ANG	31	(12.8)	19	(18.3)	9	(10.6)	3	(5.6)	
**Comorbidities** ^b^									
Absence of comorbidities	121	(48.8)	54	(50.5)	46	(52.9)	21	(38.9)	.24
Joint disease	34	(13.7)	8	(7.5)	12	(13.8)	14	(25.9)	**.01**
Cardiovascular disease^f^	62	(25.0)	18	(16.8)	23	(26.4)	21	(38.9)	**.01**
Neurologic disease	9	(3.6)	7	(6.5)	1	(1.1)	1	(1.9)	.10*
Diabetes mellitus	32	(12.9)	14	(13.1)	7	(8.0)	11	(20.4)	.10
Other diseases^g^	27	(10.9)	13	(12.1)	8	(9.2)	6	(11.1)	.80

^a^Groups were compared using the chi-square test, two-sided P-value corresponds to the comparison of the proportions between the groups recovered, mildly affected, and highly affected, classified in 2017; Significant P-values are indicated in bold (p ≤ .05).

^b^Socio-demographic characteristics measured in 2015

^c^Total recovered group n = 106, total mildly affected group n = 87, total highly affected group n = 54

^d^Total recovered group n = 104, total mildly affected group n = 85, total highly affected group n = 54.

^e^Antillian Guilder, 1 ANG = 0.55 United States Dollar.

^f^Cardiovascular disease group includes, hypertension and hypercholesterolemia

^g^Other diseases includes chronic lung diseases, auto-immune diseases, gastro-intestinal complaints, unspecified pain, allergies and other.

*Fisher’s exact test.

The age range in 2017 was 20–96 years, 51% were aged between 41–60 years and the total group had a mean (±SD) age of 53.90 (±14.91) years. The ratio of males to females was 0.37 (67 men and 181 women). The most common pre-existing comorbidities were cardiovascular diseases (n = 62; 25%), joint disease (n = 34; 14%), and diabetes mellitus (n = 32; 13%). There was a significant difference (p = .01) in the prevalence of joint and cardiovascular diseases between the disease status groups, with the highly affected patients reporting the highest prevalence. The statistically significant socio-demographic characteristics associated with being recovered vs. affected (mildly plus highly affected), and being mildly vs. highly affected based on the 2017 disease status classification are shown in S2 Table.

### Clinical presentation of long-term chikungunya disease

**Fig 2:**
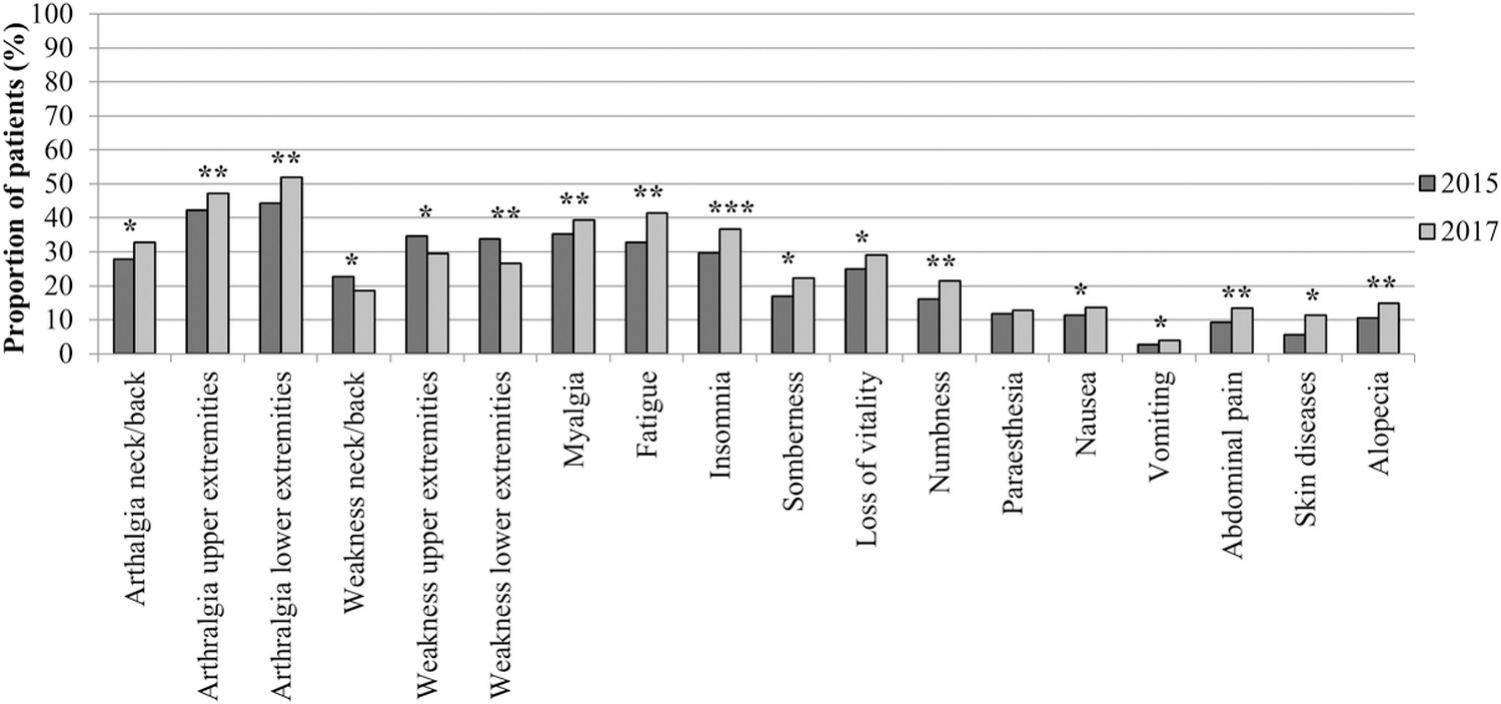
Clinical symptoms reported at the time of interview in 2015 and 2017. The horizontal axis shows the rheumatic and non-rheumatic symptoms measured in both 2015 and 2017. The proportion of symptomatic patients reporting ongoing symptoms at the time of interview are shown in the vertical axis. *P ≤ .05; **P < .01; ***P < .001.

Almost all symptoms, both rheumatic and non-rheumatic, except for joint weakness and paraesthesia, were significantly (p ≤ .05) more frequent reported by the cohort in 2017 than in 2015 (Fig 2).

### Clinical presentation reported in 2015 vs. 2017 based on the 2015 disease status classification

In 2017, the highly affected patients reported the highest prevalence of symptoms, followed by the mildly affected and the recovered patients. However, the recovered patients reported a significant higher prevalence of rheumatic symptoms including arthralgia in the neck/back (p = .03), upper (p = .002) and lower extremities (p < .001), weakness in the upper extremities (p = .006), and non-rheumatic symptoms including insomnia (p = .002), and fatigue (p = .009) in 2017 compared to 2015. The mildly affected patients reported no significant difference in rheumatic symptoms prevalence in 2017 compared to 2015. However, the mildly affected patients reported a significant higher prevalence of non-rheumatic symptoms skin diseases (p = .04) and sombreness (p = .049) in 2017 compared to 2015. In addition, the highly affected patients reported a significant lower prevalence of rheumatic symptoms arthralgia in the upper extremities (p = .03) and weakness in the upper and lower extremities (p < .001) in 2017 compared to 2015. The highly affected patients reported no significant difference in non-rheumatic symptoms prevalence in 2017 compared to 2015. The total data on the clinical symptoms based on the 2015 disease status classification are provided in S3 Table.

### Clinical presentation reported in 2015 vs. 2017 based on the 2017 disease status classification

In 2017, the recovered patients reported less prevalence of symptoms compared to the affected patients (S4 Table). The mildly affected patients reported a significant higher prevalence of ongoing rheumatic symptoms including arthralgia in the upper extremities (p = .005) and myalgia (p = .03) and the non-rheumatic/psychological symptoms fatigue (p = .04), insomnia (p = .03), and sombreness (p = .049) in 2017 compared to 2015. The highly affected patients reported a significant higher prevalence of ongoing arthralgia in the lower extremities (p = .01) and fatigue (p = .049) in 2017 compared to 2015.

In addition, the most prominent ongoing rheumatic symptoms reported by the highly affected patients were arthralgia in the upper extremities, weakness in the upper and lower extremities, myalgia, and the non-rheumatic/psychological symptoms insomnia, loss of vitality, sombreness, and a high prevalence of headache.

The statistically significant ongoing symptoms associated with being recovered vs. affected (mildly plus highly affected), and being mildly vs. highly affected based on the 2017 disease status classification are shown in S5 Table.

### Nature of symptoms and relapse frequency

**Fig 3:**
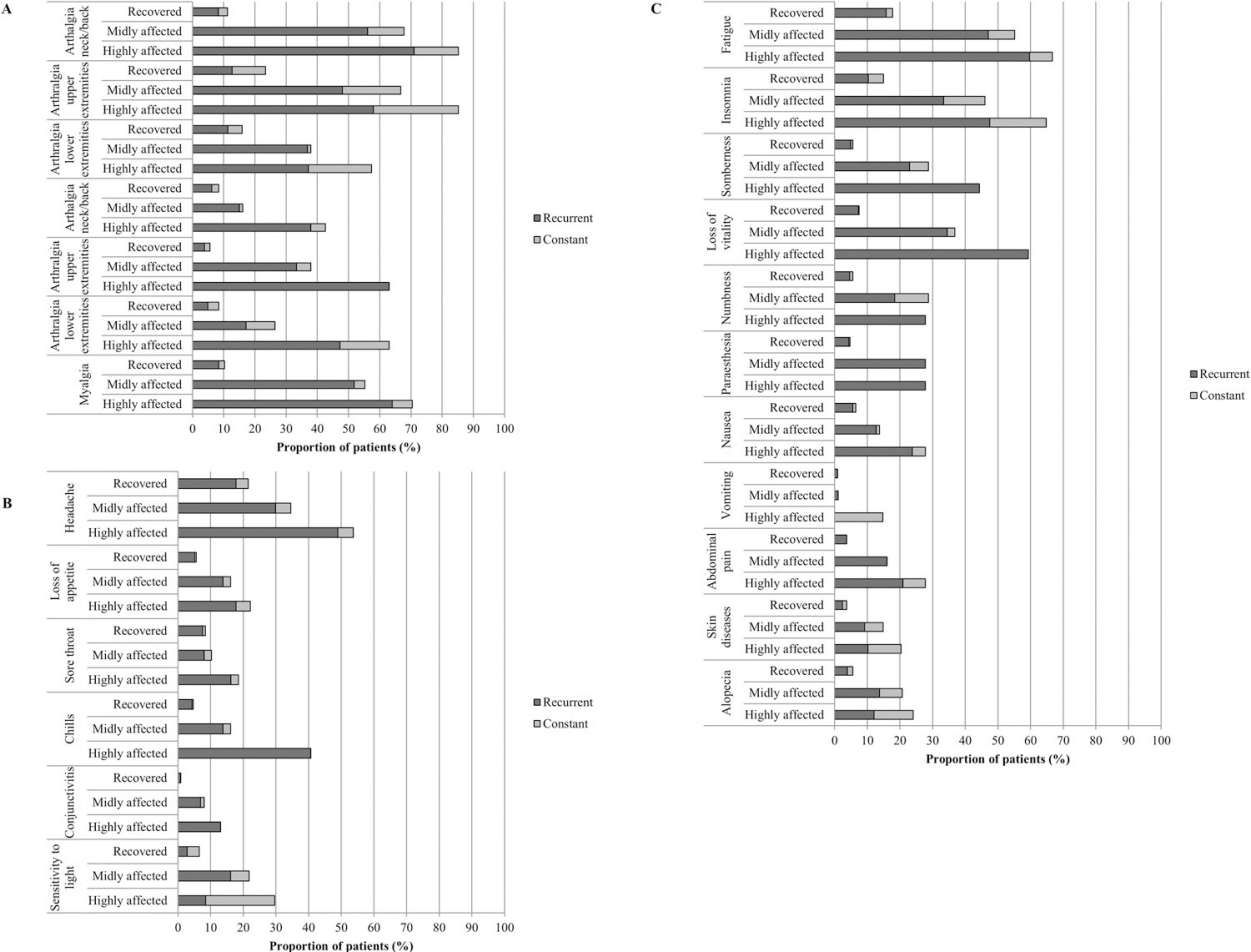
The nature of reported symptoms in 2017. The horizontal axis shows the proportion of reported symptoms at the time of interview in 2017, divided in their recurrent or constant nature. The rheumatic and non-rheumatic symptoms per group are shown in the vertical axis. Total recovered n = 107, mildly affected n = 87, and highly affected n = 54, classified in 2017. (A) Rheumatic symptoms since 2015; (B) Non-rheumatic symptoms measured since 2015; (C) Non-rheumatic symptoms measured only in 2017.

The symptoms were prominently recurrent in nature, ranging from 54.3–100% in the groups classified as affected in 2017. The most recurrent symptoms reported among the highly affected patients were rheumatic symptoms including arthralgia in the upper and lower extremities, weakness in the upper extremities, myalgia, followed by non-rheumatic/psychological symptoms fatigue, insomnia, loss of vitality, and sombreness (Fig 3A–3C).

The symptoms relapse frequency between 2015–2017 was higher among the highly affected patients. In addition, the highly affected patients reported more frequent (>20) relapses (n = 22; 51.2%), compared to the mildly affected patients (n = 15; 17.2%) (Table 3).

**Table 3:** Frequency of symptom relapses between 2015 and 2017.

	Recovered^a^		Mildly affected^b^		Highly affected^c^	
	n = 107		n = 87		n = 54	
	n	(%)	n	(%)	N	(%)
None	86	(86.0)	12	(20.3)	1	(2.3)
1–10 relapses	12	(12.0)	27	(45.8)	14	(32.5)
10–20 relapses	1	(1.0)	5	(8.5)	6	(14.0)
>20 relapses	1	(1.0)	15	(25.4)	22	(51.2)

^a^Total recovered group n = 100

^b^Total mildly affected group n = 59

^c^Total highly affected group n = 43, classified in 2017. Groups were compared using Fisher’s exact test, two-sided P-value < .001 corresponds to the comparison of the proportions of symptom relapse frequency between the recovered and affected groups.

### Chikungunya disease intensity score

**Fig 4:**
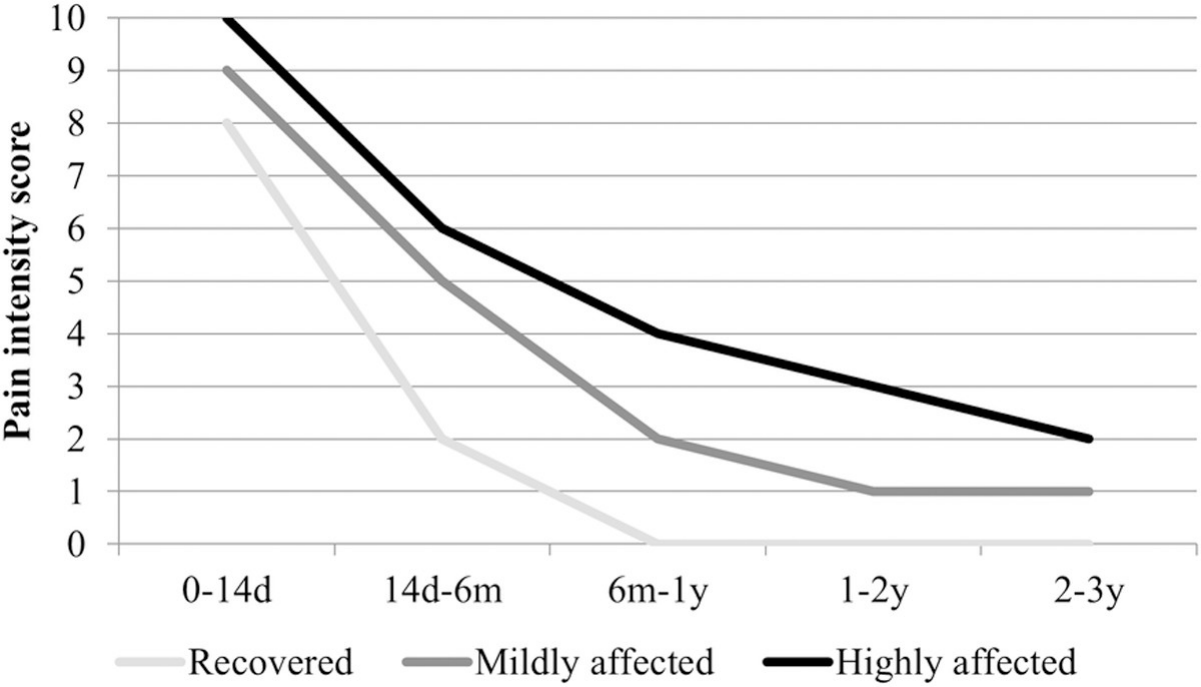
Chikungunya disease pain intensity scores. The horizontal axis shows the different time intervals, between disease onset in 2014–2015 (0d) and 2–3 years after disease onset in 2017. The vertical axis shows the median pain intensity visual analogue scale score (0 = no complaints at all; 10 = worst possible complaints I can imagine) of the recovered n = 107, mildly affected n = 87, and highly affected n = 54 groups, classified in 2017. Groups were compared using the chi-square test, two-sided P-value < .001. Abbreviations: d, day; m, months; y, years.

The majority of the patients classified as recovered and affected in 2017 reported having experienced severe pain intensity (VAS 7–10) up to 14 days after disease onset, which substantially decreased between 14 days and 6 months after disease onset. The pain intensity of 87 (81.3%) patients of the recovered group diminished gradually to no pain 6 months-1 year after disease onset. In the mildly and the highly affected groups, 62 (71.3%) and 40 (74.1%) patients, respectively, were still experiencing mild pain intensity (VAS 1–3) 2 years after disease onset (S6 Table). The highly affected patients reported an overall higher pain intensity, which remained stable with minimal improvement beyond 2 years after disease onset (Fig 4).

### Explanatory variables associated with long-term chikungunya disease status 2.5 years after disease onset

The explanatory variables with a P-value in the univariate analysis smaller than or equal to 0.1 that were included in the multivariate binary logistic regression model along with gender and age for the outcome variables recovered vs. affected (mildly plus highly) 2.5 years after disease onset were the comorbidities joint disease, cardiovascular diseases, and neurological disease, and all clinical symptoms, except sore throat. The explanatory variables along with gender and age for the outcome variables mildly vs. highly affected 2.5 years after disease onset were the comorbidities joint disease and diabetes mellitus, and all clinical symptoms, except fatigue, numbness, skin disease, alopecia, loss of appetite, sore throat, eye infection, and sensitivity to light. The explanatory variables are shown in S2 and S5 Tables.

Being recovered was associated with weakness in the back/neck (OR: 0.39; CI: 0.18–0.87; p = .02) and neurological disease (OR: 0.02; CI: 0.002–0.33; p = .005). Suffering from arthralgia in the upper (OR: 3.32; CI: 2.03–5.45; p < .001) and lower (OR: 2.44; CI: 1.58–3.76; p < .001) extremities, myalgia (OR: 2.02; CI: 1.24–3.27; p = .004) and loss of vitality (OR: 3.35; CI: 1.76–6.39; p < .001) were independently associated with being affected by chikungunya 2.5 years after disease onset (S7 Table). Being highly affected (n = 54) was associated with weakness in the lower extremities (OR: 1.90; CI: 1.29–2.80, p = .001) compared to mildly affected patients (S8 Table).

### Health-related QoL

In both 2015 and 2017, the 2017 classified recovered and mildly affected patients showed higher SF-36 QoL scores in all QoL domains, compared to the highly affected patients. In addition, the recovered, mildly affected, and highly affected patients differed significantly in all QoL domains (p ≤ .008; Kruskal Wallis test) (S9 and S10 Tables).

**Fig 5:**
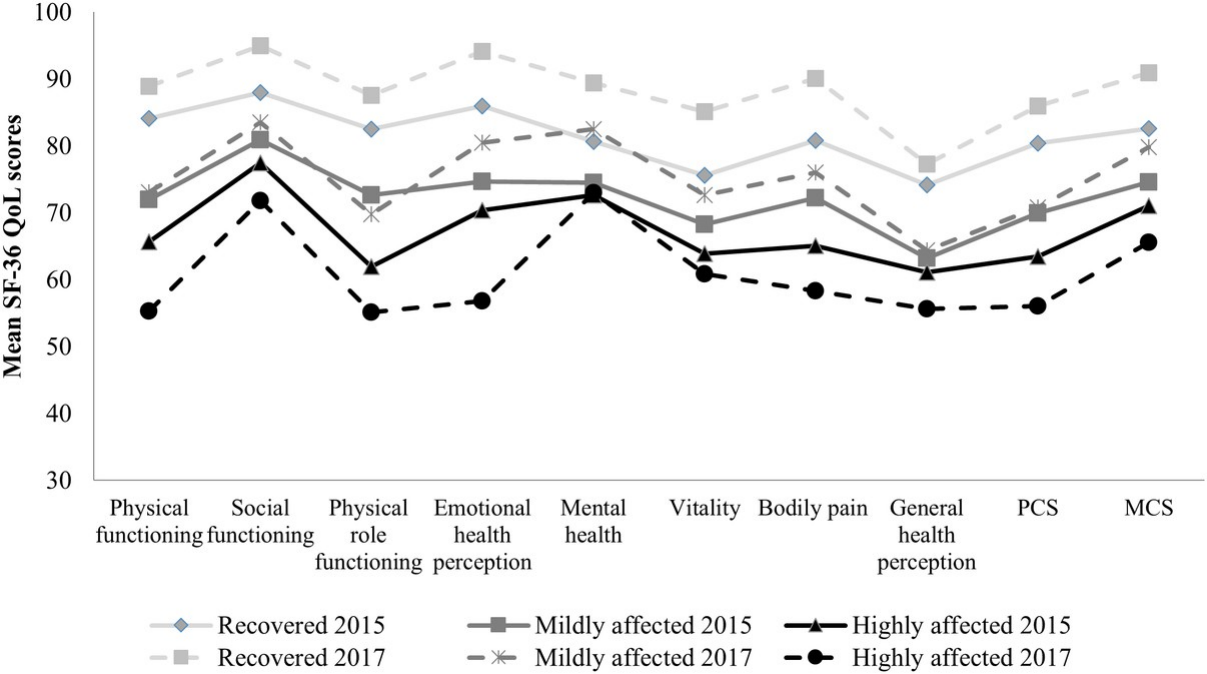
Mean 36-item short-form health survey quality of life (QoL) scores according to long-term chikungunya disease status (n = 248). The 2015 and 2017 QoL comparison of the recovered n = 107, mildly affected n = 87, and highly affected n = 54 groups, classified in 2017.

Furthermore, when evaluating the association between the 2015 and 2017 QoL scores and disease status classification in 2017, the recovered group reported an improvement of all QoL domains scores, PCS and MCS scores in 2017 compared to 2015. The mildly affected patients reported an improvement of overall QoL domains, but worsened physical role functioning and improved MCS score, with 2.9 and 5.2 points change, respectively, in 2017 compared to 2015. The highly affected patients reported an overall worsened QoL domains score, and worsened PCS and MSC scores, with 5.5 and 7.4 points change, respectively, in 2017 compared to 2015 (Fig 5).

## Discussion

This follow-up cohort study shows the progression of disease and impact of long-term sequelae on QoL 2.5 years after chikungunya disease onset, in Curaçao. We showed that rheumatic and underreported non-rheumatic/psychological symptoms (especially fatigue, insomnia, sombreness, and loss of vitality) have a major impact on QoL following chikungunya infection, and we identified the clinical profile of highly affected patients prone to develop severe QoL impairment.

Furthermore, we present the CLTCS-score, as a potential, easy to use classification instrument to characterize chronic chikungunya disease, and identify highly affected patients who are at risk of developing psychological impairment.

To our knowledge, this is the first prospective chikungunya disease cohort study that highlights the major impact of a wide range of underreported non-rheumatic in addition to rheumatic symptoms on SF-36 QoL 2.5 years following chikungunya disease onset, while most longitudinal studies solely focus on rheumatic symptoms. In 2017, 2.5 years after disease onset, more than half of our study population was classified by the CLTCS-score as being still affected by long-term chikungunya disease, resembling the high proportions of persistent rheumatic symptoms reported in other studies [9, 10, 12, 21–23].

It is expected that over the course of the disease, chikungunya-related symptoms would decrease, until full recovery [5]. However, after our baseline study in 2015 (3–16 months after disease onset), chikungunya disease remarkably progressed and a higher symptom prevalence was reported in patients classified as being affected by the CLTCS-score. In contrast to other studies [5, 13], the patients who were classified as affected in 2017 reported an increase of rheumatic symptoms especially arthralgia in the upper and/or lower extremities, myalgia, and non-rheumatic/psychological symptoms, including fatigue, insomnia, loss of vitality, and sombreness in 2017 compared to 2015, and a high prevalence of headache. The reason for the persistence of rheumatic symptoms is still unclear [24–26]. The emergence of non-rheumatic/psychological symptoms in especially the highly affected patients might be explained by the recurrent nature of symptoms and the perception of having no predictable end in sight [9, 13, 27, 28].

Indeed, the nature of the reported rheumatic and non-rheumatic symptoms in the affected groups was most commonly recurrent, as reported by other studies [7, 9, 21, 22], with at least 20 relapses between 2015 and 2017 in patients classified as highly affected in 2017.

Our results indicate that patients reporting more relapses experience the disease as worse, corresponding with a higher CLTCS-score. Interestingly, 30.1% of the patients that were classified as being recovered in 2015, were classified as being mildly or highly affected based on self-declaration in 2017. The patients classified as recovered in 2015 reported a higher prevalence of both rheumatic and non-rheumatic/psychological symptoms in 2017 compared to 2015. The fluctuation of either persistence or absence of monthly reported rheumatic complaints by affected patients over a 2 year period study has been reported [12]. The fluctuations in our study of perceived recovery in 2015 and being affected during follow-up in 2017 are most likely due to the recurrence of symptoms persistent at the time of the follow-up interview, but absent during baseline, emphasize the difficulty of possible prediction of healing [13], leading to subsequent comorbid psychological distress [9]. Raising awareness about the recurrent nature of symptoms, and the implementation of positive coping strategies interventions, may increase the ability of patients to adapt to the burden of long-term disease emotionally [28]. In addition, we also conclude that the long-term disease status of chikungunya patients could be re-assessed during follow-ups due to possible symptoms recurrence.

The patients that were classified as highly affected in 2017 reported the highest pain intensity directly after disease onset, which gradually improved significantly from severe to mild intensity over time. However, the reported pain intensity is seemingly lower compared to other studies, which may reflect the use of different recruitment criteria [23, 29]. Nevertheless, it is generally accepted that the intensity of chronic pain has a negative effect on psychological distress and QoL [30, 31].

However, in our study it was evident that even though the pain intensity decreased, a higher prevalence of psychological distress was reported, suggesting that other factors, including the persistence, increase, and symptom recurrence contributed just as much to the burden of chikungunya disease. Long-term affected individuals are currently a neglected population, in terms of both the efficacy and utility of treatment regimens [25]. While effective treatments to reduce the intensity of rheumatic symptoms are pending, clinicians should be motivated to screen for psychological symptoms, to minimize the impact of potential psychological impairment, even if the intensity of pain decreases.

Being affected by chikungunya 2.5 years after acute disease onset was independently associated with arthralgia in the upper and lower extremities, myalgia, and loss of vitality. Furthermore, being highly affected was associated with long-term persistence of weakness in the lower extremities. The persistence of rheumatic symptoms may be directly associated to disease severity [32, 33] and initial local joint inflammation during acute disease [22], as also has been reported with other arthritogenic alphaviruses [34, 35].

Previous studies of long-term affected chikungunya patients have demonstrated joint space narrowing in the distal joints and persistent arthritis of these joints following infection [29, 36, 37], suggesting that inflammation during acute disease increases the probability of persistent rheumatic symptoms. Thus, clinical management of acute CHIKV disease symptoms and inflammation may reduce the morbidity and impact of long-term chikungunya disease. In addition, gender [11, 21], older age [21, 26, 38], and pre-existing comorbidities including joint diseases and cardiovascular diseases [39] have been reported to play a role in predisposing individuals to the severe clinical presentation of chikungunya disease and non-recovery. However, in contrast to these studies, but in accordance with others [8, 40–42], we did not find any association between gender, age, and pre-exciting comorbidities with being long-term affected by chikungunya disease in the multivariate analyses.

Similar to what was found in our baseline study conducted in 2015 and in other studies [8–10, 12, 13], the PCS, MCS, and overall QoL in 2017 were more impaired, when patients were more affected by chikungunya disease. In addition, a drop of 7.4 and 5.5 point for the PCS and MCS scores of the patients classified as highly affected in 2017 was seen, suggesting that the burden of disease has an increasing negative impact on both the physical and mental well-being of these patients over time. Interestingly, the decrease in pain intensity in the highly affected patients did not result in an improved PCS or MCS.

Our findings show that patients reporting a higher symptom prevalence or frequent relapses, regardless of a diminished pain intensity, have an increased occurrence of both physical disability and psychological comorbidity, and are more susceptible to PCS and MCS QoL impairment. Clinicians should be vigilant as psychological symptoms and MCS impairment can be difficult to recognize [43, 44], and therefore may remain untreated. Subsequently, reliable and easy to use instruments like the CLTCS-score are needed in the assessment of long-term disease impact, to facilitate in identifying patients at risk for developing QoL impairment. In addition, our previous [8] and current studies show that the performance of the CLTCS-score proves to be consistent over time and the categorization can be linked to all aspects of chikungunya disease burden. Being categorized as highly affected by the CLTCS-score, corresponds with a higher prevalence of persistent symptoms of recurrent nature, higher frequency of symptom relapses and pain intensity, and the degree of QoL impairment.

Our study has several strengths and limitations. Among its strengths is the fact that the study population is composed of individuals representative for the socio-economic characteristics of the general population. However, patients with more severe disease were perhaps more likely to respond and to participate. Nevertheless, the response rate of the follow-up study was 82.5%, limiting selection bias. This study conducted a prospective evaluation of persistent symptoms, which minimizes the chance of recall bias and an overestimation of experienced symptoms. Furthermore, most studies define long-term chikungunya disease by the criterion of ‘arthralgia’ persistency, which may result in an underestimation of long-term disease, while the CLTCS-score used in our study captures the broader range of the clinical presentation of chikungunya disease taking the recurrent nature of symptoms into account. A limitation of this study was the gender imbalance, with a male female ratio of 0.37, which may restrict the generalizability of the findings [21, 45, 46]. Further limitations were the absence of data of possibly new developed comorbidities between the 2015 and 2017 studies (although comorbidities as reported in baseline study were used in analyses) and no rheumatological examinations. Furthermore, no healthy control group was recruited. However, there is a significant difference of persistent symptoms between the recovered and affected groups. In addition, the whole study population was not tested for other arthritogenic arbovirus, like dengue and Zika. Though, unlike CHIKV, dengue and Zika viruses are not known to cause long-term arthralgia [47].

In conclusion, this study highlights the need for long-term patient follow-up. Long-term chikungunya disease poses a major yet often underestimated burden on affected patients. Long-term chikungunya disease should not be characterized merely as the persistence of debilitating polyarthralgia, but should also include the underreported comorbid psychological impairment.

The knowledge about the impact of both rheumatic and non-rheumatic/psychological symptoms of long-term chikungunya disease, as presented in this study, is imperative in the context of developing multidisciplinary interventions, which should be focused on improving functional ability, emotional and mental response, to minimize QoL impairment in highly affected patients. In addition, the CLTCS-score can facilitate health care providers in research and clinical settings to easily identify highly affected patients who are at risk of developing psychological impairment.

Using these findings for interventions educating the public on the risk of severe physical and psychological complications of long-term disease can be an effective public health approach particularly in regions where the prevalence of long-term disease is rising.

## Supporting information

S1 Strobe checklist Checklist for cohort study. (PDF)

S1 Table Overview of the 2017 follow-up study participants (n = 248). (PDF)

S2 Table Univariate analysis of socio-demographic characteristics comparing the recovered vs. affected and mildly affected vs. highly affected groups, based on the disease status classification in 2017 (n = 248). (PDF)

S3 Table Univariate analysis of clinical presentation reported in 2015 and 2017 by long-term chikungunya disease status classification in 2015 (n = 248). (PDF)

S4 Table Univariate analysis of clinical presentation reported in 2015 and 2017 by long-term chikungunya disease status classification in 2017 (n = 248). (PDF)

S5 Table Univariate analysis of clinical presentation reported in 2017 comparing the recovered vs. affected and mildly affected vs. highly affected groups, based on the disease classification in 2017 (n = 248). (PDF)

S6 Table Chikungunya disease intensity score of symptoms between disease onset and 2–3 years after disease onset comparing the recovered, mildly affected, and highly affected groups, based on the disease status classification in 2017. (PDF)

S7 Table Final model of variables associated with being defined as long-term affected vs. recovered from chikungunya disease 2.5 years after disease onset. (PDF)

S8 Table Final model of variables associated with being defined as highly affected vs. mildly affected by long-term chikungunya disease 2.5 years after disease onset. (PDF)

S9 Table The 2015 SF-36 QoL scores among the 2017 recovered, mildly affected, and highly affected groups. (PDF)

S10 Table The 2017 SF-36 QoL scores among the 2017 recovered, mildly affected, and highly affected groups. (PDF)
