# Correction: Long-term Chikungunya sequelae and quality of life 2.5 years post-acute disease in a prospective cohort in Curaçao

**DOI:** 10.1371/journal.pntd.0011359

**Published:** 2023-05-19

**Authors:** The PLOS Neglected Tropical Diseases Staff

There is an error in the Author Summary section that affects the understanding of the results and conclusion of the paper. The word physiological should be psychological. Physiological relates to the chemical or biological processes of the body, which we did not measure, whereas, psychological relates to the processes of the mind, which is one of our main assessments.
