# Supplementary material for: Long-term Chikungunya sequelae and quality of life 2.5 years post-acute disease in a prospective cohort in Curaçao
Source: PLoS Negl Trop Dis. 2022 Mar 1;16(3):e0010142. doi: 10.1371/journal.pntd.0010142 (PMC8887759; doi:10.1371/journal.pntd.0010142)
Supplement: S1 Strobe checklist — (PDF) [file pntd.0010142.s001.pdf]

STROBE Statement—checklist of items that should be included in reports of observational studies

	Item No.	Recommendation	Relevant text from manuscript
**Title and abstract**	1	(*a*) Indicate the study's design with a commonly used term in the title or the abstract	The title states ''prospective cohort''
		(*b*) Provide in the abstract an informative and balanced summary of what was done and what was found	This was provided in the Abstract and Author summary sections
**Introduction**			
Background/rationale	2	Explain the scientific background and rationale for the investigation being reported	This was explained in the Introduction section; paragraph 3
Objectives	3	State specific objectives, including any pre-specified hypotheses	This was stated in the Introduction section; paragraph 6
**Methods**			
Study design	4	Present key elements of study design early in the paper	This was presented in the Methods section under the sub-section '' Study design and population''
Setting	5	Describe the setting, locations, and relevant dates, including periods of recruitment, exposure, follow-up, and data collection	This was described in the Methods section under the sub-sections ''Setting'', ''Study design and population'', ''Baseline assessment'', ''Follow-up and clinical presentation'', and ''Health related QoL''
Participants	6	(*a*) *Cohort study*—Give the eligibility criteria, and the sources and methods of selection of participants. Describe methods of follow-up<br>*Case-control study*—Give the eligibility criteria, and the sources and methods of case ascertainment and control selection. Give the rationale for the choice of cases and controls<br>*Cross-sectional study*—Give the eligibility criteria, and the sources and methods of selection of participants	This was given in the Methods section under the sub-sections '' Study design and population'' and ''Follow-up and clinical presentation''
		(*b*) *Cohort study*—For matched studies, give matching criteria and number of exposed and unexposed<br>*Case-control study*—For matched studies, give matching criteria and the number of controls per case	Not applicable

Continued on next page

Variables	7	Clearly define all outcomes, exposures, predictors, potential confounders, and effect modifiers. Give diagnostic criteria, if applicable	This was defined in the Method section under the sub-section ‘’Data analysis’’
Data sources/ measurement	8*	For each variable of interest, give sources of data and details of methods of assessment (measurement). Describe comparability of assessment methods if there is more than one group	This was given in the Method section under the sub-sections ‘’Baseline assessment’’, ‘’Follow-up and clinical presentation’’, and ‘’Health related QoL’’
Bias	9	Describe any efforts to address potential sources of bias	This was described in the Methods section under sub-section ‘’Follow-up and clinical presentation’’
Study size	10	Explain how the study size was arrived at	This was explained in the Methods section under sub-section ‘’Study design and population’’
Quantitative variables	11	Explain how quantitative variables were handled in the analyses. If applicable, describe which groupings were chosen and why	This was explained in the Methods section under sub-section ‘’Data analysis’’; paragraph 2
Statistical methods	12	(*a*) Describe all statistical methods, including those used to control for confounding	This was described in the Methods section under sub-section ‘’Data analysis’’; paragraphs 2 and 3
		(*b*) Describe any methods used to examine subgroups and interactions	This was described in the Methods section under sub-section ‘’Data analysis’’; paragraph 1
		(*c*) Explain how missing data were addressed	This was explained in the Methods section under sub-section ‘’Data analysis’’; paragraph 2
		(*d*) *Cohort study*—If applicable, explain how loss to follow-up was addressed<br>*Case-control study*—If applicable, explain how matching of cases and controls was addressed<br>*Cross-sectional study*—If applicable, describe analytical methods taking account of sampling strategy	This was explained in the Methods section under sub-section ‘’Follow-up and clinical presentation’’
		(*e*) Describe any sensitivity analyses	Not applicable
**Results**			
Participants	13*	(a) Report numbers of individuals at each stage of study—eg numbers potentially eligible, examined for eligibility, confirmed eligible, included in the study, completing follow-up, and analysed	This was reported in the Results section; paragraph 1
		(b) Give reasons for non-participation at each stage	This was given in the Results section; paragraph 1

Continued on next page

		(c) Consider use of a flow diagram		Not applicable
Descriptive data	14*	(a) Give characteristics of study participants (eg demographic, clinical, social) and information on exposures and potential confounders	12-13	This was given in the Results section under sub-section ‘’Long-term disease status and characteristics study population’’; paragraphs 1 and 2
		(b) Indicate number of participants with missing data for each variable of interest	12-21	This was indicated in the Results section tables footnotes
		(c) *Cohort study*—Summarise follow-up time (eg, average and total amount)	12	This was summarized in the Results section; paragraph 1
Outcome data	15*	*Cohort study*—Report numbers of outcome events or summary measures over time	12-21	This was reported in the Results section under sub-sections ‘’Long-term disease status and characteristics study population’’, ‘’Clinical presentation of long-term chikungunya disease’’, ‘’Prognostic factors associated with long-term chikungunya disease status’’, and ‘’Health related QoL’’
		Case-control study—Report numbers in each exposure category, or summary measures of exposure		Not applicable
		Cross-sectional study—Report numbers of outcome events or summary measures		Not applicable
Main results	16	(*a*) Give unadjusted estimates and, if applicable, confounder-adjusted estimates and their precision (eg, 95% confidence interval). Make clear which confounders were adjusted for and why they were included	19-20	This was given in the Results section under sub-section ‘’Explanatory variables associated with long-term chikungunya disease status 2.5 years after disease onset’’
		(*b*) Report category boundaries when continuous variables were categorized		Not applicable
		(*c*) If relevant, consider translating estimates of relative risk into absolute risk for a meaningful time period		Not applicable
Other analyses	17	Report other analyses done—eg analyses of subgroups and interactions, and sensitivity analyses		Not applicable

Continued on next page

**Discussion**			
Key results	18	Summarise key results with reference to study objectives	This was summarized in the Discussion section; paragraph 1
Limitations	19	Discuss limitations of the study, taking into account sources of potential bias or imprecision. Discuss both direction and magnitude of any potential bias	This was discussed in the Discussion section; paragraph 9
Interpretation	20	Give a cautious overall interpretation of results considering objectives, limitations, multiplicity of analyses, results from similar studies, and other relevant evidence	This was given in the Discussion section; paragraph 8
Generalisability	21	Discuss the generalisability (external validity) of the study results	This was discussed in the Discussion section; paragraphs 8 and 10
**Other information**			
Funding	22	Give the source of funding and the role of the funders for the present study and, if applicable, for the original study on which the present article is based	This was given in the funding statement

*Give information separately for cases and controls in case-control studies and, if applicable, for exposed and unexposed groups in cohort and cross-sectional studies.

Note: An Explanation and Elaboration article discusses each checklist item and gives methodological background and published examples of transparent reporting. The STROBE checklist is best used in conjunction with this article (freely available on the Web sites of PLoS Medicine at http://www.plosmedicine.org/, Annals of Internal Medicine at http://www.annals.org/, and Epidemiology at http://www.epidem.com/). Information on the STROBE Initiative is available at www.strobe-statement.org.
