# Supplementary material for: Long-term Chikungunya sequelae and quality of life 2.5 years post-acute disease in a prospective cohort in Curaçao
Source: PLoS Negl Trop Dis. 2022 Mar 1;16(3):e0010142. doi: 10.1371/journal.pntd.0010142 (PMC8887759; doi:10.1371/journal.pntd.0010142)
Supplement: S1 Table — (PDF) [file pntd.0010142.s002.pdf]

	n
**Cohort participants 2015, with laboratory confirmation**	304
**Non-contacting reasons 2017 follow-up study**	
Participant was not reached by phone nor house-visits after 3 failed attempts	10
Participant has died	4
Participant was abroad or on holiday	4
Non-participant circumstances prevented interviewer from (completing) interviewing	3
**Non-response reasons 2017 follow-up study**	
Participant refused	20
Participant not interviewed after contact, due to circumstances	15
**Consenting cohort participants 2017 follow-up study**	**248**
