# Supplementary material for: Long-term Chikungunya sequelae and quality of life 2.5 years post-acute disease in a prospective cohort in Curaçao
Source: PLoS Negl Trop Dis. 2022 Mar 1;16(3):e0010142. doi: 10.1371/journal.pntd.0010142 (PMC8887759; doi:10.1371/journal.pntd.0010142)
Supplement: S2 Table — (PDF) [file pntd.0010142.s003.pdf]

	Recovered (n = 107)		Affected (n = 141)			Mildly affected (n = 87)		Highly affected (n = 54)		
	**n**	**(%)**	**n**	**(%)**	**P-value[a]**	**n**	**(%)**	**n**	**(%)**	**P-value[a]**
**Gender**					**.04**					.64
Female	71	(66.4)	110	(78.0)		69	(79.3)	41	(75.9)	
Male	36	(33.6)	31	(22.0)		18	(20.7)	13	(24.1)	
**Age (years)**					.10					.50
20-40	25	(23.4)	19	(13.5)		14	(16.1)	5	(9.3)	
41-60	49	(45.8)	78	(55.3)		46	(52.9)	32	(59.3)	
≥61	33	(30.8)	44	(31.2)		27	(31.0)	17	(31.5)	
**Education**					.54					.17
Illiterate/primary school	26	(24.3)	32	(22.7)		16	(18.4)	16	(29.6)	
Secondary school	36	(33.6)	51	(36.2)		33	(37.9)	18	(33.3)	
Intermediate vocational education	25	(23.4)	40	(28.4)		29	(33.3)	11	(20.4)	
University (of applied sciences)	20	(18.7)	18	(12.8)		9	(10.3)	9	(16.7)	
**Occupation**[bc]					.43					.30
Unemployed/student/housewife/voluntary	16	(15.1)	31	(22.0)		21	(24.1)	10	(18.5)	
Paid job (domestic or manual)	51	(48.1)	55	(39.0)		32	(36.8)	23	(42.6)	
Paid job (not domestic or manual)	21	(19.8)	29	(20.6)		21	(24.1)	8	(14.8)	
Retired	18	(17.0)	26	(18.4)		13	(14.9)	13	(24.1)	
**Income**[bde]					.11					.73*
0-999 ANG	10	(9.6)	13	(9.4)		7	(8.2)	6	(11.1)	
1000-2499 ANG	42	(40.4)	55	(39.6)		34	(40.0)	21	(38.9)	
2500-4999 ANG	33	(31.7))	59	(42.4)		35	(41.2)	24	(44.4)	
≥5000 ANG	19	(18.3	12	(8.6)		9	(10.6)	3	(5.6)	
**Comorbidities**[b]										
Absence of comorbidities	54	(50.5)	67	(47.5)	.65	46	(52.9)	21	(38.9)	.11
Joint disease	8	(7.5)	26	(18.4)	**.01**	12	(13.8)	14	(53.8)	.07
Cardiovascular disease[f]	18	(16.8)	44	(31.2)	**.01**	23	(26.4)	21	(38.9)	.12
Neurologic disease	7	(6.5)	2	(1.4)	**.04***	1	(1.1)	1	(1.9)	1.000*
Diabetes mellitus	14	(13.1)	18	(12.8)	.94	7	(8.0)	11	(20.4)	**.03**
Other diseases[g]	13	(12.1)	14	(9.9)	.58	8	(9.2)	6	(11.1)	.71

[a]Groups were compared using the chi-square test, two-sided P-value corresponds to the comparison of the proportions between the recovered and affected (mildly affected plus highly affected) groups, classified in 2017; Significant P-values are indicated in bold ($p \leq .05$). [b]Socio-demographic characteristics measured in 2015. [c]Total recovered group n = 106, total affected group n = 141; [d]Total recovered group n = 104 and total affected

group n = 139, total mildly affected group n = 85 and total highly affected group n = 54. [e]Antillian Guilder, 1 ANG = 0.61 USD. [f]Cardiovascular disease group includes, hypertension and hypercholesterolemia; [g]Other diseases, includes chronic lung diseases, auto-immune diseases, gastro-intestinal complaints, unspecified pain, allergies and other. *Fisher's exact test.
