# Supplementary material for: Long-term Chikungunya sequelae and quality of life 2.5 years post-acute disease in a prospective cohort in Curaçao
Source: PLoS Negl Trop Dis. 2022 Mar 1;16(3):e0010142. doi: 10.1371/journal.pntd.0010142 (PMC8887759; doi:10.1371/journal.pntd.0010142)
Supplement: S3 Table — (PDF) [file pntd.0010142.s004.pdf]

	Recovered (n = 93)					Mildly affected (n = 84)					Highly affected (n = 71)				
	2015		2017			2015		2017			2015		2017		
	n	(%)	n	(%)	P-value[a]	n	(%)	n	(%)	P-value[a]	n	(%)	n	(%)	P-value[a]
**Arthralgia in the**															
neck/back	8	(8.6)	19	(20.4)	**.03**	30	(35.7)	29	(34.5)	1.000	31	(43.7)	33	(46.5)	.85
upper extremities[b]	7	(7.5)	24	(25.8)	**.002**	40	(47.6)	48	(54.8)	.39	58	(81.7)	47	(66.2)	**.03**
lower extremities[c]	9	(9.7)	30	(32.3)	**< .001**	41	(48.8)	46	(54.8)	.52	60	(84.5)	53	(74.6)	.17
**Weakness in the**															
neck/back	7	(7.5)	12	(12.9)	.27	24	(28.6)	15	(17.9)	.09	25	(35.2)	19	(26.8)	.33
upper extremities[b]	2	(2.2)	12	(12.9)	**.006**	32	(38.1)	29	(34.5)	.74	52	(73.2)	32	(45.1)	**< .001**
lower extremities[c]	7	(7.5)	13	(14.0)	.18	26	(31.0)	24	(28.6)	.86	51	(71.8)	29	(40.8)	**< .001**
**Myalgia**[d]	8	(8.6)	19	(20.4)	**.01**	36	(42.9)	38	(45.2)	.87	43	(61.4)	40	(57.1)	.72
**Fatigue**	13	(14.0)	27	(29.0)	**.009**	25	(29.8)	33	(39.3)	.15	43	(60.6)	43	(60.6)	1.000
**Insomnia**	10	(10.8)	26	(28.0)	**.002**	24	(28.6)	27	(32.1)	.65	40	(56.3)	38	(53.5)	.86
**Sombreness**[d]	3	(3.2)	11	(11.8)	**.02**	11	(13.1)	20	(23.8)	**.049**	28	(40.0)	23	(32.9)	.36
**Loss of vitality**	7	(7.5)	18	(19.4)	**.01**	17	(20.2)	22	(26.2)	.38	38	(53.5)	32	(45.1)	.29
**Numbness**	0	(0.0)	8	(8.6)	1.000	14	(16.7)	18	(21.4)	.52	26	(36.6)	27	(38.0)	1.000
**Paraesthesia**	2	(2.2)	6	(6.5)	.29	9	(10.7)	9	(10.7)	1.000	18	(25.4)	17	(23.9)	1.000
**Nausea**	1	(1.1)	9	(9.7)	**.008**	10	(11.9)	13	(15.5)	.58	17	(23.9)	12	(16.9)	.36
**Vomiting**[e]	0	(0.0)	1	(1.1)	1.000	5	(6.0)	4	(4.8)	1.000	2	(2.8)	5	(7.0)	.45
**Abdominal pain**[f]	2	(2.2)	6	(6.5)	.22	8	(9.5)	9	(10.7)	1.000	13	(18.3)	18	(25.4)	.36
**Skin diseases**	4	(4.3)	5	(5.4)	1.000	2	(2.4)	10	(11.9)	**.04**	8	(11.3)	13	(18.3)	.30
**Alopecia**	4	(4.3)	7	(7.5)	.55	7	(8.3)	13	(15.5)	.15	15	(21.1)	17	(23.9)	.82
**Headache**[†]			26	(28.0)				24	(28.6)				32	(45.1)	
**Loss of appetite**[†]			9	(9.7)				6	(7.1)				17	(23.9)	
**Sore throat**[†]			9	(9.7)				8	(9.5)				11	(15.5)	
**Chills**[†]			10	(10.8)				16	(19.0)				15	(21.1)	
**Eye infection**[†]			2	(2.2)				4	(4.8)				9	(12.7)	

**Sensitivity to light**[†]	15	(16.1)	13	(15.5)	14	(19.7)

[a]Clinical symptoms were compared using the McNemar test, two-sided P-value corresponds to the comparison of the proportions of participants answering ‘yes’ (participants answering ‘somewhat’ and ‘yes’ summed) and those responding ‘no’ among the groups recovered, mildly affected, and highly affected, classified in 2015; Significant P-values are indicated in bold ($p \leq .05$). [b]Upper extremities refers to the shoulders, hands, wrists, and fingers; [c]Lower extremities refers to the hips, knees, ankles, feet, and toes; [e]Total highly affected group n = 70; [e]Total mildly affected group n = 83; [f]Total recovered group n = 92. [†]Non-rheumatic symptoms (headache, loss of appetite, sore throat, chills, conjunctivitis, and sensitivity to light) that were only measured in 2017.
