# Supplementary material for: Long-term Chikungunya sequelae and quality of life 2.5 years post-acute disease in a prospective cohort in Curaçao
Source: PLoS Negl Trop Dis. 2022 Mar 1;16(3):e0010142. doi: 10.1371/journal.pntd.0010142 (PMC8887759; doi:10.1371/journal.pntd.0010142)
Supplement: S4 Table — (PDF) [file pntd.0010142.s005.pdf]

	Recovered (n = 107)					Mildly affected (n = 87)					Highly affected (n = 54)				
	2015		2017			2015		2017			2015		2017		
	n	(%)	n	(%)	P-value[a]	n	(%)	n	(%)	P-value[a]	n	(%)	n	(%)	P-value[a]
**Arthralgia in the**															
neck/back	18	(16.8)	17	(15.9)	1.000	26	(29.9)	33	(37.9)	.25	25	(46.3)	31	(57.4)	.31
upper extremities[b]	23	(21.5)	12	(11.2)	.06	42	(48.3)	59	(67.8)	**.005**	40	(74.1)	46	(85.2)	.26
lower extremities[c]	29	(27.1)	25	(23.4)	.59	46	(52.9)	58	(66.7)	.07	35	(64.8)	46	(85.2)	**.01**
**Weakness in the**															
neck/back	15	(14.0)	9	(8.4)	.21	19	(21.8)	14	(16.1)	.41	22	(40.7)	23	(42.6)	1.000
upper extremities[b]	16	(15.0)	6	(5.6)	**.04**	35	(40.2)	33	(37.9)	.87	35	(64.8)	34	(63.0)	1.000
lower extremities[c]	20	(18.7)	9	(8.4)	**.04**	36	(41.4)	23	(26.4)	.06	28	(51.9)	34	(63.0)	.24
**Myalgia**[d]	20	(18.9)	11	(10.4)	.09	32	(36.8)	48	(55.2)	**.03**	35	(64.8)	38	(70.4)	.63
**Fatigue**	19	(17.8)	19	(17.8)	1.000	35	(40.2)	48	(55.2)	**.04**	27	(50.0)	36	(66.7)	**.049**
**Insomnia**	18	(16.8)	16	(15.0)	.85	28	(32.2)	40	(46.0)	**.03**	28	(51.9)	35	(64.8)	.17
**Sombreness**[e]	9	(8.4)	6	(5.6)	.58	16	(18.4)	25	(28.7)	**.049**	17	(32.1)	23	(43.4)	.21
**Loss of vitality**	11	(10.3)	8	(7.5)	.61	27	(31.0)	32	(36.8)	.46	24	(44.4)	32	(59.3)	.08
**Numbness**	8	(7.5)	6	(5.6)	.77	17	(19.5)	25	(28.7)	.15	15	(27.8)	22	(40.7)	.14
**Paraesthesia**	4	(3.7)	5	(4.7)	1.000	13	(14.9)	12	(13.8	1.000	12	(13.8)	15	(27.8)	.63
**Nausea**	4	(3.7)	7	(6.5)	.51	13	(14.9)	12	(13.8)	1.000	11	(20.4)	15	(27.8)	.48
**Vomiting**[f]	2	(1.9)	1	(0.9)	1.000	2	(2.3)	1	(1.2)	1.000	3	(5.6)	8	(14.8)	.13
**Abdominal pain**[f]	3	(2.8)	4	(3.7)	1.000	10	(11.6)	14	(16.3)	.42	10	(18.5)	15	(27.8)	.30
**Skin diseases**	3	(2.8)	4	(3.7)	1.000	5	(5.7)	13	(14.9)	.08	6	(11.1)	11	(20.4)	.23
**Alopecia**	7	(6.5)	6	(5.6)	1.000	11	(12.6)	18	(20.7)	.06	8	(14.8)	13	(24.1)	.30
**Headache**[†]			23	(21.5)				30	(34.5)				29	(53.7)	
**Loss of appetite**[†]			6	(5.6)				14	(16.1)				12	(22.2)	
**Sore throat**[†]			9	(8.4)				9	(10.3)				10	(18.5)	
**Chills**[†]			5	(4.7)				14	(16.1)				22	(40.7)	
**Eye infection**[†]			1	(0.9)				7	(8.0)				7	(13.0)	
**Sensitivity to light**[†]			7	(6.5)				19	(21.8)				16	(29.6)	

[a]Clinical symptoms were compared using the McNemar test, two-sided P-value corresponds to the comparison of the proportions of participants answering ‘yes’ (participants answering ‘somewhat’ and ‘yes’ summed) and those responding ‘no’ among the groups recovered, mildly affected, and highly affected, classified in 2017; Significant P-values are indicated in bold ($p \leq .05$). [b]Upper extremities refers to the shoulders, hands, wrists, and fingers; [c]Lower extremities refers to the hips, knees, ankles, feet, and toes; [d]Total recovered group n = 106; [e]Total highly affected group n = 53; [f]Total mildly affected group n = 86. [†]Non-rheumatic symptoms (headache, loss of appetite, sore throat, chills, conjunctivitis, and sensitivity to light) that were only measured in 2017. *Fisher’s exact test.
