# Supplementary material for: Long-term Chikungunya sequelae and quality of life 2.5 years post-acute disease in a prospective cohort in Curaçao
Source: PLoS Negl Trop Dis. 2022 Mar 1;16(3):e0010142. doi: 10.1371/journal.pntd.0010142 (PMC8887759; doi:10.1371/journal.pntd.0010142)
Supplement: S5 Table — (PDF) [file pntd.0010142.s006.pdf]

	Recovered (n = 107)				Affected (n = 141)					Mildly affected (n = 87)				Highly affected (n = 54)				
	No		Yes		No		Yes			No		Yes		No		Yes		
	n	(%)	n	(%)	n	(%)	n	(%)	P-value[a]	n	(%)	n	(%)	n	(%)	n	(%)	P-value[a]
**Arthralgia in the**																		
neck/back	90	(84.1)	17	(15.9)	77	(54.6)	64	(45.4)	**< .001**	54	(62.1)	33	(37.9)	23	(42.6)	31	(57.4)	**.02**
upper extremities[b]	95	(88.8)	12	(11.2)	36	(25.5)	105	(47.2)	**< .001**	28	(32.2)	59	(67.8)	8	(14.8)	46	(85.2)	**.02**
lower extremities[c]	82	(76.6)	25	(23.4)	37	(26.2)	104	(73.8)	**< .001**	29	(33.3)	58	(66.7)	8	(14.8)	46	(85.2)	**.02**
**Weakness in the**																		
neck/back	98	(91.6)	9	(8.4)	104	(73.8)	37	(26.2)	**< .001**	73	(83.9)	14	(16.1)	31	(57.4)	23	(42.6)	**.001**
upper extremities[b]	101	(94.4)	6	(5.6)	74	(52.5)	67	(47.5)	**< .001**	54	(62.1)	33	(37.9)	20	(37.0)	34	(63.0)	**.004**
lower extremities[c]	98	(91.6)	9	(8.4)	84	(59.6)	57	(40.4)	**< .001**	64	(73.6)	23	(26.4)	20	(37.0)	34	(63.0)	**< .001**
**Myalgia**	96	(89.7)	11	(10.3)	55	(39.0)	86	(61.0)	**< .001**	39	(44.8)	48	(55.2)	16	(29.6)	38	(70.4)	.07
**Fatigue**	88	(82.2)	19	(17.8)	57	(40.4)	84	(59.6)	**< .001**	39	(44.8)	48	(55.2)	18	(33.3)	36	(66.7)	.18
**Insomnia**	91	(85.0)	16	(15.0)	66	(46.8)	75	(53.2)	**< .001**	47	(54.0)	40	(46.0)	19	(35.2)	35	(64.8)	**.03**
**Sombreness**	101	(94.4)	6	(5.6)	92	(65.2)	49	(34.8)	**< .001**	62	(71.3)	25	(28.7)	30	(55.6)	24	(44.4)	**.06**
**Loss of vitality**	99	(92.5)	8	(7.5)	77	(54.6)	64	(45.4)	**< .001**	55	(63.2)	32	(36.8)	22	(40.7)	32	(59.3)	**.009**
**Numbness**	101	(94.4)	6	(5.6)	94	(66.7)	47	(33.3)	**< .001**	62	(71.3)	25	(28.7)	32	(59.3)	22	(40.7)	.14
**Paraesthesia**	102	(95.3)	5	(4.7)	114	(80.9)	27	(19.1)	**.001**	75	(86.2)	12	(13.8)	39	(72.2)	15	(27.8)	**.04**

**Nausea**	100	(93.5)	7	(6.5)	114	(80.9)	27	(19.1)	**.004**	75	(86.2)	12	(13.8)	39	(72.2)	15	(27.8)	**.04**
**Vomiting**	106	(99.1)	1	(0.9)	132	(93.6)	9	(6.4)	**.046***	86	(98.9)	1	(1.1)	46	(85.2)	8	(14.8)	**.002***
**Abdominal pain**	103	(96.3)	4	(3.7)	112	(79.4)	29	(20.6)	**< .001**	73	(83.9)	14	(16.1)	39	(72.2)	15	(27.8)	.10
**Skin diseases**	103	(96.3)	4	(3.7)	117	(83.0)	24	(17.0)	**.001**	74	(85.1)	13	(14.9)	43	(79.6)	11	(20.4)	.40
**Alopecia**	101	(94.4)	6	(5.6)	110	(78.0)	31	(22.0)	**< .001**	69	(79.3)	18	(20.7)	41	(75.9)	13	(24.1)	.64
**Headache**	84	(78.5)	23	(21.5)	82	(58.2)	59	(41.8)	**.001**	57	(65.5)	30	(34.5)	25	(46.3)	29	(53.7)	**.03**
**Loss of appetite**	101	(94.4)	6	(5.6)	115	(81.6)	26	(18.4)	**.003**	73	(83.9)	14	(16.1)	42	(77.8)	12	(22.2)	.36
**Sore throat**	98	(91.6)	9	(8.4)	122	(86.5)	19	(13.5)	.21	78	(89.7)	9	(10.3)	44	(81.5)	10	(18.5)	.17
**Chills**	102	(95.3)	5	(4.7	105	(74.5)	36	(25.5)	**< .001**	73	(83.9)	14	(16.1)	32	(59.3)	22	(40.7)	**.001**
**Eye infection**	106	(99.1)	1	(0.9)	127	(90.1)	14	(9.9)	**.003**	80	(92.0)	7	(8.0)	47	(87.0)	7	(13.0)	.34
**Sensitivity to light**	100	(93.5)	7	(6.5)	106	(75.2)	35	(24.8)	**< .001**	68	(78.2)	19	(21.8)	38	(70.4)	16	(29.6)	.30

[a]Groups were compared using the chi-square test, two-sided P-value corresponds to the comparison of the proportions of participants answering ‘no’ or ‘yes (somewhat and yes)’ between the recovered and affected (mildly affected plus highly affected) groups, classified in 2017; Significant P-values are indicated in bold ($p \leq .05$). [b]Upper extremities refers to the shoulders, hands, wrists, and fingers; [c]Lower extremities refers to the hips, knees, ankles, feet, and toes. *Fisher’s exact test.
