# Supplementary material for: Long-term Chikungunya sequelae and quality of life 2.5 years post-acute disease in a prospective cohort in Curaçao
Source: PLoS Negl Trop Dis. 2022 Mar 1;16(3):e0010142. doi: 10.1371/journal.pntd.0010142 (PMC8887759; doi:10.1371/journal.pntd.0010142)
Supplement: S6 Table — (PDF) [file pntd.0010142.s007.pdf]

| | Recovered (n = 107) | | Mildly affected (n = 87) | | Highly affected (n = 54) | | |
|---|---|---|---|---|---|---|---|
| | n | (%) | n | (%) | n | (%) | P-value[a] |
| **Intensity complaints day 0-14** | | | | | | | < .001* |
| *No pain* | 2 | (1.9) | 0 | (0.0) | 0 | (0.0) | |
| *Mild pain* | 14 | (13.1) | 1 | (1.1) | 0 | (0.0) | |
| *Moderate pain* | 22 | (20.6) | 3 | (3.4) | 5 | (9.3) | |
| *Severe pain* | 69 | (64.5) | 83 | (95.4) | 49 | (90.7) | |
| **Intensity complaints day 14-6 months** | | | | | | | < .001 |
| *No pain* | 39 | (36.4) | 2 | (2.3) | 0 | (0.0) | |
| *Mild pain* | 31 | (29.0) | 14 | (16.1) | 9 | (16.7) | |
| *Moderate pain* | 32 | (29.9) | 43 | (49.4) | 25 | (46.3) | |
| *Severe pain* | 5 | (4.7) | 28 | (32.2) | 20 | (37.0) | |
| **Intensity complaints 6 months-1 year** | | | | | | | < .001* |
| *No pain* | 87 | (81.3) | 14 | (16.1) | 0 | (0.0) | |
| *Mild pain* | 16 | (15.0) | 51 | (58.6) | 26 | (48.1) | |
| *Moderate pain* | 4 | (3.7) | 17 | (19.5) | 24 | (44.4) | |
| *Severe pain* | 0 | (0.0) | 5 | (5.7) | 4 | (7.4) | |
| **Intensity complaints 1-2 year** | | | | | | | < .001* |
| *No pain* | 105 | (98.1) | 18 | (20.7) | 0 | (0.0) | |
| *Mild pain* | 1 | (0.9) | 64 | (73.6) | 36 | (66.7) | |
| *Moderate pain* | 1 | (0.9) | 3 | (3.4) | 15 | (27.8) | |
| *Severe pain* | 0 | (0.0) | 2 | (2.3) | 3 | (5.6) | |
| **Intensity complaints 2-3 year** | | | | | | | <. 001* |
| *No pain* | 105 | (98.1) | 22 | (25.3) | 0 | (0.0) | |
| *Mild pain* | 2 | (1.9) | 62 | (71.3) | 40 | (74.1) | |
| *Moderate pain* | 0 | (0.0) | 2 | (2.3) | 10 | (18.5) | |
| *Severe pain* | 0 | (0.0) | 1 | (1.1) | 4 | (7.4) | |

[a]Groups were compared using the chi-square test, two-sided P-value corresponds to the comparison of the proportions of patients experiencing no pain (visual analogue scale <1), mild pain (visual analogue scale 1-3), moderate pain (visual analogue scale 4-6), and severe pain (visual analogue scale 7-10) between the recovered and affected groups, classified in 2017. *Fisher's exact test.
