# Supplementary material for: Long-term Chikungunya sequelae and quality of life 2.5 years post-acute disease in a prospective cohort in Curaçao
Source: PLoS Negl Trop Dis. 2022 Mar 1;16(3):e0010142. doi: 10.1371/journal.pntd.0010142 (PMC8887759; doi:10.1371/journal.pntd.0010142)
Supplement: S7 Table — (PDF) [file pntd.0010142.s008.pdf]

**Variable**	**Odds Ratio (OR)**	**95% CI**	**P-value**
**Arthralgia in the upper extremities**			
No	Reference	Reference	
Yes (somewhat/yes)	3.32	2.03-5.45	**< .001**
**Arthralgia in the lower extremities**			
No	Reference	Reference	
Yes (somewhat/yes)	2.44	1.58-3.76	**< .001**
**Weakness in the back/neck**			
No	Reference	Reference	
Yes (somewhat/yes)	0.39	0.18-0.87	**.02**
**Myalgia**			
No	Reference	Reference	
Yes (somewhat/yes)	2.02	1.24-3.27	**.004**
**Loss of vitality**			
No	Reference	Reference	
Yes (somewhat/yes)	3.35	1.76-6.39	**< .001**
**Neurologic disease**			
No	Reference	Reference	
Yes	0.02	0.002-0.33	**.005**
