# Supplementary material for: Long-term Chikungunya sequelae and quality of life 2.5 years post-acute disease in a prospective cohort in Curaçao
Source: PLoS Negl Trop Dis. 2022 Mar 1;16(3):e0010142. doi: 10.1371/journal.pntd.0010142 (PMC8887759; doi:10.1371/journal.pntd.0010142)
Supplement: S8 Table — (PDF) [file pntd.0010142.s009.pdf]

Variable	Odds Ratio (OR)	95% CI	P-value
**Weakness in the lower extremities**			
No	Reference	Reference	
Yes (somewhat/yes)	1.90	1.29-2.79	**.001**
