# Supplementary material for: Long-term Chikungunya sequelae and quality of life 2.5 years post-acute disease in a prospective cohort in Curaçao
Source: PLoS Negl Trop Dis. 2022 Mar 1;16(3):e0010142. doi: 10.1371/journal.pntd.0010142 (PMC8887759; doi:10.1371/journal.pntd.0010142)
Supplement: S9 Table — (PDF) [file pntd.0010142.s010.pdf]

	Recovered (n = 107)		Mildly affected (n = 87)		Highly affected (n = 54)		
	**Median (IQR)**	**Mean (SD)**	**Median (IQR)**	**Mean (SD)**	**Median (IQR)**	**Mean (SD)**	**P-value[a]**
**Physical functioning**	100 (70.0-100)	84.1 (24.9)	80.0 (50.0-100)	72.0 (25.8)	70.0 (45.0-90.0)	65.7 (29.1)	< .001
**Social functioning**	100 (75.0-100)	88.0 (17.3)	87.5 (62.5-100)	80.9 (20.7)	87.5 (62.5-100)	77.5 (23.0)	.002
**Physical role functioning**	100 (100-100)	82.5 (34.9)	100 (50.0-100)	72.7 (39.4)	100 (0.0-100)	62.0 (44.4)	.005
**Emotional health perception**	100 (100-100)	86.0 (33.3)	100 (66.7-100)	74.7 (40.3)	100 (25.0-100)	70.4 (42.3)	.008
**Mental health**	84.0 (76.0-92.0)	80.7 (14.8)	80.0 (64.0-88.0)	74.5 (16.5)	80.0 (63.0-88.0)	72.7 (20.4)	.003
**Vitality**	80.0 (65.0-90.0)	75.6 (18.4)	70.0 (55.0-80.0)	68.3 (17.2)	65.0 (50.0-80.0)	63.9 (20.2)	< .001
**Bodily pain**	89.8 (67.3-100)	80.8 (21.5)	67.3 (57.1-89.8)	72.2 (23.2)	67.3 (44.9-79.6)	65.1 (23.2)	< .001
**General health perception**	80.0 (65.0-85.0)	74.2 (15.9)	65.0 (50.0-75.0)	63.2 (18.3)	62.5 (45.0-80.0)	61.1 (20.2)	< .001
**PCS[b]**	87.5 (73.7-93.8)	80.4 (19.0)	76.3 (53.1-87.5)	70.0 (21.7)	72.1 (41.7-86.2)	63.5 (24.8)	< .001
**MCS[c]**	87.5 (78.1-94.8)	82.6 (16.7)	82.9 (60.2-89.5)	74.6 (19.9)	78.2 (55.9-90.0)	71.1 (22.8)	< .001

[a]Two-sided P-value obtained using Kruskal-Wallis test; SF-36 scores from 0 (worst) to 100 (best). [b]Physical component summary (PCS) includes the domains Physical functioning, Physical role functioning, Bodily pain, and General health perception. [c]Mental component summary (MCS) includes the domains Social functioning, Emotional health perception, Mental health, and Vitality.
