# Supplementary material for: Long-term Chikungunya sequelae and quality of life 2.5 years post-acute disease in a prospective cohort in Curaçao
Source: PLoS Negl Trop Dis. 2022 Mar 1;16(3):e0010142. doi: 10.1371/journal.pntd.0010142 (PMC8887759; doi:10.1371/journal.pntd.0010142)
Supplement: S10 Table — (PDF) [file pntd.0010142.s011.pdf]

	Recovered (n = 107)		Mildly affected (n = 87)		Highly affected (n = 54)		
	**Median (IQR)**	**Mean (SD)**	**Median (IQR)**	**Mean (SD)**	**Median (IQR)**	**Mean (SD)**	**P-value[a]**
**Physical functioning**	100 (90.0-100)	88.9 (21.8)	80.0 (55.0-95.0)	73.1 (24.0)	50.0 (33.8-85.0)	55.3 (30.9)	< .001
**Social functioning**	100 (100-100)	95.0 (11.8)	87.5 (62.5-100)	83.5 (20.6)	75.0 (62.5-100)	71.8 (26.1)	< .001
**Physical role functioning**	100 (100-100)	87.6 (30.8)	100 (25.0-100)	69.8 (43.5)	75.0 (0.0-100)	55.1 (47.2)	< .001
**Emotional health perception**	100 (100-100)	94.1 (22.3)	100 (100-100)	80.5 (37.9)	66.7 (0.0-100)	56.8 (45.6)	< .001
**Mental health**	92.0 (84.0-100)	89.4 (13.2)	84.0 (72.0-96.0)	82.5 (16.6)	78.0 (64.0-88.0)	73.0 (20.3)	< .001
**Vitality**	90.0 (75.0-100)	85.1 (15.0)	75.0 (60.0-85.0)	72.7 (18.0)	62.5 (48.8-75.0)	60.9 (20.7)	< .001
**Bodily pain**	100 (87.8-100)	90.1 (17.1)	79.6 (67.4-89.8)	76.0 (19.2)	66.3 (44.9-79.6)	58.3 (22.9)	< .001
**General health perception**	80.0 (70.0-90.0)	77.3 (14.9)	70.0 (50.0-75.0)	64.4 (17.7)	57.5 (43.8-75.0)	55.6 (23.0)	< .001
**PCS[b]**	92.5 (83.5-96.3)	86.0 (16.6)	77.4 (49.3-87.5)	70.8 (21.1)	57.1 (32.9-81.1)	56.1 (26.3)	< .001
**MCS[c]**	94.0 (88.3-100)	90.9 (12.4)	86.9 (67.9-95.0)	79.8 (19.3)	73.0 (43.8-87.7)	65.6 (25.0)	< .001

[a]Two-sided P-value obtained using Kruskal-Wallis test; SF-36 scores from 0 (worst) to 100 (best). [b]Physical component summary (PCS) includes the domains Physical functioning, Physical role functioning, Bodily pain, and General health perception. [c]Mental component summary (MCS) includes the domains Social functioning, Emotional health perception, Mental health, and Vitality.
